# The novel mitochondria-targeted hydrogen sulfide (H_2_S) donors AP123 and AP39 protect against hyperglycemic injury in microvascular endothelial cells *in vitro*

**DOI:** 10.1016/j.phrs.2016.08.019

**Published:** 2016-11

**Authors:** Domokos Gerő, Roberta Torregrossa, Alexis Perry, Alicia Waters, Sophie Le-Trionnaire, Jacqueline L. Whatmore, Mark Wood, Matthew Whiteman

**Affiliations:** a University of Exeter Medical School, Exeter, UK; b Biosciences, College of Life and Environmental Sciences, University of Exeter, UK; c IRSET-UMR INSERM U1085, Equipe 3-Stress, Membrane et Signalisation, Rennes Cedex, France

**Keywords:** Hydrogen sulfide, Oxidative stress, Electron transport, Superoxide, Hyperglycemia, Endothelial cells, Bioenergetics, Complex II, SQR

## Abstract

The development of diabetic vascular complications is initiated, at least in part, by mitochondrial reactive oxygen species (ROS) production in endothelial cells. Hyperglycemia induces superoxide production in the mitochondria and initiates changes in the mitochondrial membrane potential that leads to mitochondrial dysfunction. Hydrogen sulfide (H_2_S) supplementation has been shown to reduce the mitochondrial oxidant production and shows efficacy against diabetic vascular damage *in vivo.* However, the half-life of H_2_S is very short and it is not specific for the mitochondria. We have therefore evaluated two novel mitochondria-targeted anethole dithiolethione and hydroxythiobenzamide H_2_S donors (AP39 and AP123 respectively) at preventing hyperglycemia-induced oxidative stress and metabolic changes in microvascular endothelial cells *in vitro*. Hyperglycemia (HG) induced significant increase in the activity of the citric acid cycle and led to elevated mitochondrial membrane potential. Mitochondrial oxidant production was increased and the mitochondrial electron transport decreased in hyperglycemic cells. AP39 and AP123 (30–300 nM) decreased HG-induced hyperpolarisation of the mitochondrial membrane and inhibited the mitochondrial oxidant production. Both H_2_S donors (30–300 nM) increased the electron transport at respiratory complex III and improved the cellular metabolism. Targeting H_2_S to mitochondria retained the cytoprotective effect of H_2_S against glucose-induced damage in endothelial cells suggesting that the molecular target of H_2_S action is within the mitochondria. Mitochondrial targeting of H_2_S also induced >1000-fold increase in the potency of H_2_S against hyperglycemia-induced injury. The high potency and long-lasting effect elicited by these H_2_S donors strongly suggests that these compounds could be useful against diabetic vascular complications.

## Graphical abstract

**Figure:**
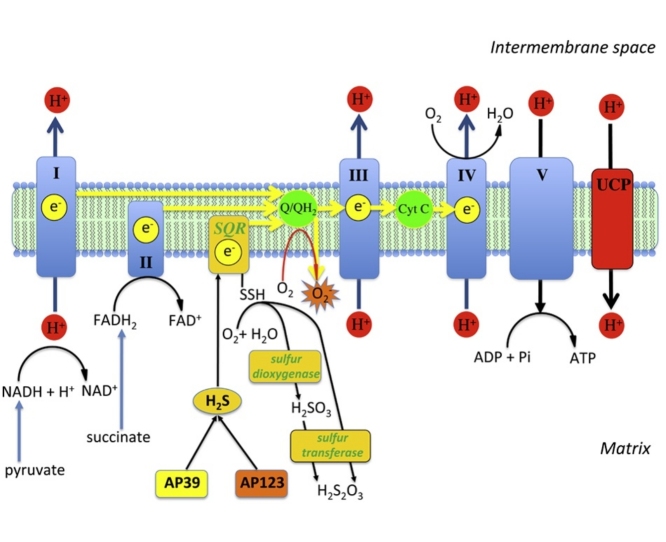

## 1 Introduction

Diabetic complications are responsible for the majority of expenses associated with diabetes treatment and the costs of diabetes that currently accounts for 10% of total healthcare costs, is projected to increase to 17% of health resource expenditure over the next 20 years [1]. Diabetes diagnostic criteria were established based on the increased risk of microvascular and cardiovascular complications in patients with increased plasma glucose level [2] and glycemic control represent the foundation of diabetes therapy, still it provides little protection against cardiovascular disease (CVD) [3]. Since glucose control is ineffective against cardiovascular events in diabetic patients [3], [4], [5], it is important to find novel therapies that reduce the progression of cardiovascular disease in diabetes. Hyperglycemia induces oxidant production in the vessels and oxidative stress is considered as a major contributor to vascular damage [6]. Oxidative stress induced by hyperglycemia persists in the cells long after glucose levels are normalised and this phenomenon is known as “glucose memory” [7]. The limited CVD risk reduction in diabetes may be explained by the persistence of deleterious downstream effects that occur after intermittent hyperglycemic episodes, despite lower glycated hemoglobin levels.

Hyperglycemia-induced mitochondrial superoxide generation is an upstream player in the development of endothelial dysfunction and it is responsible for the activation of other sources of oxidants in the cells [8]. In endothelial cells, high glucose supply results in increased glucose oxidation: more electron donors are pushed into the electron transport chain and the voltage gradient across the mitochondrial membrane increases. The increased transmembrane voltage induces electron leakage between complexes II and III and the inappropriate transfer of electrons to molecular oxygen generates superoxide [9]. If the mitochondrial potential is normalised in the cells by uncoupling protein-1 (UCP-1) overexpression or the mitochondrial respiratory chain is inactivated by mitochondrial DNA depletion hyperglycemia does not generate superoxide [8]. Blockage of mitochondrial superoxide generation by the above methods or neutralisation by manganese superoxide dismutase (MnSOD) inhibits other sources of oxidants in endothelial cells: the activation of protein kinase C (PKC) and the polyol pathway, the formation of advanced glycation end product (AGE) and the hexosamine pathway [8]. Similarly, we found that mitochondrial superoxide scavenging using paroxetine [10] or induction of uncoupling protein-2 (UCP-2) also blocked the glucose-induced oxidant production in endothelial cells [11]. While these methods all reduce the mitochondrial ROS production and the associated cellular damage, neither ROS scavenging nor mitochondrial uncoupling fully restore the mitochondrial energy production. If electrons are used for superoxide generation and the protons are released through uncoupling proteins, there will be a drop in ATP production *via* oxidative phosphorylation.

H_2_S is an endogenously produced ‘gasotransmitter’ that plays key roles in regulating vascular tone, inflammation, cell death and proliferation as well as vascular protection [12], [13], [14], [15]. Lower H_2_S bioavailability has been reported in the diabetic vasculature in humans and correlates to poorer microcirculatory blood flow [16]. Impaired vascular H_2_S synthesis and/or bioavailability is also observed in the vasculature of several animal models of diabetes induced either pharmacologically- (*e.g.* streptozotocin-induced [17]) or genetically-induced (*e.g.* Akita [18], db/db [19] and NOD mice [20]). The ‘loss’ of vasculoprotective H_2_S is thought to contribute to vascular endothelial dysfunction and disease pathology suggesting approaches to increase H_2_S bioavailability could be of therapeutic benefit in diabetes and vascular disease. One key mechanism by which H_2_S is beneficial is by serving as an inorganic electron donor to the respiratory chain [21]. The oxidation of H_2_S is a multi-step process and electron transfer to the respiratory chain may be dissociated from the subsequent steps of proton transfer and oxygen consumption [22]. Thus, unlike the main electron donors, nicotinamide adenine dinucleotide (NADH) and flavin adenine dinucleotide (FADH_2_), H_2_S can provide the respiratory chain with electrons only. This effect of H_2_S is supported by the findings that exogenous H_2_S, albeit at high concentrations, can normalise the mitochondrial membrane potential and reduce mitochondrial superoxide generation in hyperglycemic endothelial cells and also prevent the development of endothelial dysfunction in streptozotocin-induced diabetes [17], [23]. Furthermore, H_2_S, in the form of inorganic salts (*e.g.* NaSH and Na_2_S) have protective effects against diabetic retinopathy and nephropathy [24], [25], [26] and also has cardioprotective effects in diabetic models [19], [27], [28]. The administration of H_2_S using its sodium salts is inconvenient in long-term diseases because it has a short half-life and lacks cellular targeting. Natural sources of H_2_S such as garlic were therefore, also tested with similar results: garlic extract protects against diabetic nephropathy, vasculopathy and cardiomyopathy [29], [30], [31] and its active constituents were found to be diallyldisulfide (DADS) and diallyltrisulfide (DATS) [32], which are slower H_2_S donors than Na_2_S [33]. Since the protective effect of H_2_S is mostly mitochondrial in the hyperglycemic endothelium, we tested the efficacy of novel mitochondria-targeted H_2_S donors against the glucose-induced oxidant production. AP39 is a slow-release H_2_S donor that was shown to accumulate in the mitochondria [36], [37] and protect against oxidative stress-induced mitochondrial DNA and protein damage in endothelial cells [34]. We compared the efficacy of AP39 and AP123, a newer mitochondrial H_2_S donor, against glucose-induced endothelial dysfunction and we found that mitochondrial slow-release H_2_S donors are >1000-fold more potent than Na_2_S against hyperglycemia-induced oxidant production and also have beneficial effect on cellular bioenergetics in endothelial cells.

## 2 Methods

### 2.1 Synthesis of mitochondria-targeted H_2_S donor AP123 (10-(4-carbamothioylphenoxy)-10-oxodecyl)triphenylphosphonium bromide)

AP39 was synthesised as previously described by us [35], extinction coefficient in DMSO (λ_400nm_ = 6162 M^−1^ cm^−1^; (λ_327nm_ = 12000 M^−1^ cm^−1^). AP123 was synthesised using the following procedure: acetonitrile (8 cm^3^) was added to 10-bromodecanoic acid (400 mg, 1.59 mmol) and triphenylphosphine (418 mg, 1.59 mmol) and the resulting mixture was stirred and heated under reflux for 48 h [36]. The acetonitrile was evaporated *in vacuo* and the colourless, oily residue was triturated with toluene (3 × 10 cm^3^) before thorough drying on a rotary evaporator and dissolution in dichloromethane (15 cm^3^). At room temperature, 4-hydroxythiobenzamide (244 mg, 1.59 mmol) was added to the stirred solution, followed by a solution of *N*,*N*-dicyclohexylcarbodiimide (330 mg, 1.60 mmol) in dichloromethane (8 cm^3^) and 4-dimethylaminopyridine (10 mg, 0.08 mmol). After stirring for 22 h, the reaction mixture was filtered through a cotton wool plug and after removal of the solvent *in vacuo*, the crude product was applied as a dichloromethane solution onto a silica gel flash chromatography column *ca* 120 cm^3^ silica gel, 3 cm diameter column). After flushing the silica gel with ethyl acetate (200 cm^3^), the product was eluted with methanol (200 cm^3^) and after evaporation of the solvent *in vacuo*, the product was re-dissolved in dichloromethane (20 cm^3^) and the resulting solution was dried (magnesium sulfate), filtered and evaporated *in vacuo* to give the title compound (516 mg, 50%) as a crisp, yellow foam (found [M-Br]^+^ (ES^+^) 568.2429, C_35_H_39_NO_2_PS requires 568.2434); *ν*_max_ (KBr disc)/cm^−1^ 3415 (m), 3055 (m), 2925 (s), 2853 (s), 1752 (s) (C=O), 1619 (s), 1599 (s), 1587 (m), 1504 (m), 1483 (m), 1464 (w), 1438 (s), 1384 (m), 1311 (m), 1264 (m), 1205 (s), 1167 (s), 1112 (s), 1014 (m), 995 (m), 892 (m) and 851 (w); ^1^H NMR (300 MHz, CDCl_3_) 9.26 (1H, br s, N*H*), 8.20 (2H, part of AA'BB', *J* = 8.5 Hz, aryl C*H*), 7.89-7.62 (16H, complex, phenyl C*H* and N*H*), 7.02 (2H, part of AA'BB', *J* = 8.5 Hz, aryl C*H*), 3.50 (2H, m, C*H*_2_P^+^), 2.52, (2H, t, *J* = 7 Hz, C*H*_2_C(O)), 1.72-1.55 and 1.42-1.13 (6H and 8H, 2 x broad m, (C*H*_2_)_7_C(O)); ^31^P NMR (121 MHz, CDCl_3_) 24.0 (P^+^); ^13^C NMR (100 MHz, CDCl_3_) 200.0 (*C* = S), 171.8 (*C*=O), 153.5 (aryl *C*-O), 135.6 (aryl *C*-C(S)), 135.2 (d, *J* = 3 Hz, phenyl *C*-H), 133.5 (d, *J* = 10 Hz, 2 x phenyl *C*-H), 130.5 (d, *J* = 13 Hz, 2 x phenyl *C*-H), 129.7 (aryl *C*-H), 121.0 (aryl *C*-H), 118.1 (d, *J* = 86 Hz, phenyl *C*-P^+^), 34.2 (*C*H_2_C(O)), 30.3 (*C*H_2_), 30.1 (*C*H_2_), 28.9 (*C*H_2_), 28.8 (*C*H_2_), 28.6 (*C*H_2_), 28.5 (*C*H_2_), 24.5 (*C*H_2_), 22.9 (*C*H_2_) and 22.4 (d, *J* = 18 Hz, *C*H_2_P^+^), extinction coefficient in DMSO (λ_308 nm_ = 5275 M^−1^ cm^−1^; (λ_262 nm_ = 8108 M^−1^ cm^−1^).

### 2.2 H_2_S release detection

H_2_S donors were dissolved and diluted in DMSO. Compounds or vehicle were added in 1/10 vol and mixed with DMEM supplemented with 10% FBS and 0.5 mg/ml MTT. Free H_2_S as strong reducing agent reacts with the tetrazolium dye 3-(4,5-dimethyl-2-thiazolyl)-2,5-diphenyl-2H-tetrazolium bromide (MTT, Calbiochem, EMD BioSciences, San Diego, CA) and forms purple colour formazan. Changes in absorbance were recorded every 24 h on a microplate reader (Molecular Devices Spectramax M2e, Sunnyvale, CA) at 570 nm with background measurement at 690 nm. The reaction was carried out in a humidified incubator at 37 °C with 5% CO_2_ atmosphere to closely mimic the cell culture conditions and minimise evaporation. H_2_S calibration curve was created by preparing serial dilutions of freshly dissolved Na_2_S (Alpha Aesar, Haverhill, MA) and by measuring the reducing capacity. The slow release H_2_S donors liberate H_2_S over several days and the low background of MTT reduction allows H_2_S detection up to 2 weeks. The H_2_S generation is shown as the cumulative increase or daily change in absorbance with respective H_2_S values.

### 2.3 Cell culture and toxicity assay

b.End3 murine microvascular endothelial cells were obtained from the European Collection of Cell Cultures (ECACC, Salisbury, UK) as described, passage numbers 24–30 were used [37]. The b.End3 cells were established from brain endothelial cells of 129/Sv mice by immortalisation with the Polyoma virus middle T-antigen [37]. The cells were maintained in Dulbecco’s modified Eagle’s medium (DMEM) (Biochrom AG, Berlin, Germany) containing 1 g/l glucose supplemented with 10% fetal bovine serum (FBS, Hyclone, Logan, UT), 1% non-essential amino acids, 100 IU/ml penicillin and 100 μg/ml streptomycin (Invitrogen, Carlsbad, CA) at 37 °C in 5% CO_2_ atmosphere [10], [11].

b.END3 endothelial cells (20 000/well) were seeded in 96 well plates and cultured in DMEM containing 1 g/l glucose supplemented with 10% FBS, 1% non-essential amino acids and antibiotics at 37 °C in 5% CO_2_ atmosphere for 5 days. H_2_S donors were diluted in PBS containing 10% DMSO and added in 1/20 vol, then cells were incubated at 37 °C for 24 h. Non-mitochondrial H_2_S donors were added in the concentration range of 100 nM to 1 mM and mitochondrial H_2_S donors in the range of 10 nM to 100 μM. After 24 h, the supernatant was saved to detect LDH release and fresh culture medium supplemented with 0.5 mg/ml MTT was added to the cells. MTT and LDH assays were performed as detailed below. The cellular viability values and percent cell lysis values were plotted and the 50% toxic concentration was calculated using Prism 6 analysis software (GraphPad Software, Inc., La Jolla, CA).

### 2.4 *In situ* detection of H_2_S in endothelial cells

b.End3 cells (2 × 10^5^/well) were seeded on 4-well Nunc Lab-Tek chambered coverglass (Nalge Nunc, Rochester, NY) and cultured overnight. H_2_S donor compounds were diluted in PBS and DMSO and were added at 30 μM final concentration in 1/20 culture volume. The cells were treated with the compounds at 37 °C for 2 h, followed by loading with fluorescent H_2_S sensor 7-azido-4-methylcoumarin (AzMc) (40 nM, Sigma-Aldrich, St. Louis, MO) and Mitotracker Green FM (200 μM, Life Technologies, Carlsbad, CA) mitochondrial stain at 37 °C for 1 h to detect H_2_S release simultaneously with the endogenous H_2_S production. AzMc fluorescence and the MitoTracker signal were detected on a Nikon TE2000 inverted microscope (Nikon UK Limited, Surrey, UK) using a Hamamatsu ORCA-ER monochrome camera (Hamamatsu Photonics UK Ltd., Hertfordshire). The H_2_S signal is shown in green and the MitoTracker signal in red.

### 2.5 High glucose-induced endothelial dysfunction

Mitochondrial ROS generation was induced in b.End3 endothelial cells by prolonged exposure to high glucose as we previously described [10], [11]. Microvascular endothelial cells (20,000/well) were seeded into 96-well tissue culture plates and were cultured for 24 h. Hyperglycemia (40 mM glucose) was initiated by replacing the culture medium with fresh DMEM containing 7.2 g/l glucose supplemented with 10% FBS, 1% non-essential amino acids, 100 IU/ml penicillin and 100 μg/ml streptomycin and the cells were exposed to high glucose level for 7 days. The culture medium was supplemented with pyruvate (10 mM) as fresh source of energy after 3 days of exposure. H_2_S donor compounds were dissolved in dimethyl sulfoxide (DMSO) and dilutions were made in phosphate buffered saline (PBS) to administer the compounds in 1/20 culture volume with final DMSO concentration of 0.5%. The cells were treated with the compounds for 3 days by administering the drugs on the 4th day of the hyperglycemic exposure.

### 2.6 MTT and LDH assays

The MTT assay and LDH activity measurements were performed as previously described [38], [39]. Briefly, the cells were incubated in culture medium containing 0.5 mg/ml MTT for 1 h at 37 °C at 10% CO_2_ atmosphere. The converted formazan dye was dissolved in isopropanol and the absorbance was measured at 570 nm with background measurement at 690 nm. Absorbance values are shown as the cellular MTT conversion rate (metabolic activity) in hyperglycemic cells. Cellular viability rates are calculated using serial dilutions of cells and the percent survival rates compared to vehicle treated controls were calculated.

Total LDH content of the cells was measured by lysing the cells in 0.15 M saline containing 1% Triton-X-100 and measuring the LDH activity by adding 100 μl LDH assay reagent containing 110 mM lactic acid, 1350 mM nicotinamide adenine dinucleotide (NAD^+^), 290 mM *N*-methylphenazonium methyl sulfate (PMS), 685 mM 2-(4-iodophenyl)-3-(4-nitrophenyl)-5-phenyl-2*H*-tetrazolium chloride (INT) and 200 mM Tris (pH 8.2). The changes in absorbance were read kinetically at 492 nm for 15 min (kinetic LDH assay). LDH activity values are shown as Vmax values. In the toxicity assay, cell death was measured by LDH release in the cell culture supernatant (30 μl/well) after 24 h exposure.

### 2.7 Measurement of mitochondrial ROS production

Μeasurements of the mitochondrial superoxide generation by MitoSOX Red and the cellular reactive oxygen species (ROS) production by CM-H_2_DCFDA were previously described [10]. After the hyperglycemia exposure the cells were loaded with the mitochondrial superoxide sensor MitoSOX™ Red (2.5 μM, Life Technologies, Carlsbad, CA) or with the cell-permeable ROS indicator 5-(and-6)-chloromethyl-2′,7′-dichlorodihydrofluorescein diacetate (CM-H_2_DCFDA, 10 μM, Life Technologies, Carlsbad, CA) and DNA stain Hoechst 33342 (10 μM) for 25 min. Reading medium (PBS supplemented with 1 g/l glucose and 10% bovine growth serum (BGS, Hyclone, Logan, UT)) was added to the cells and the oxidation of MitoSOX™ Red (Ex/Em: 530/590 nm) or CM-H_2_DCFDA (Ex/Em: 485/528 nm) was recorded kinetically on Synergy 2 plate reader (BioTek, Winooski, VT) at 37 °C for 35 min. ROS production is shown as the Vmax value of the fluorescence probe oxidation or as percent values of Vmax values of control cells. The fluorescence of Hoechst 33342 (Ex/Em: 360/460 nm) was used to confirm that there was no change in the cellular viability.

### 2.8 Mitochondrial membrane potential

The mitochondrial potential was measured with JC-1 (Sigma-Aldrich, St. Louis, MO) fluorescent probe as previously described [11], [40]. The cells were loaded with the dye by exposing them to JC-1 stain solution containing 10 μM JC-1 and 0.6 mM β-cyclodextrin (Sigma-Aldrich, St. Louis, MO) in OptiMEM I medium at 37 °C for 30 min. Subsequently, the cells were washed in phosphate buffered saline (PBS) and the red (Ex/Em: 530/590 nm) and green (Ex/Em: 485/528 nm) fluorescence was measured on a microplate reader (Synergy 2, Biotek, Winooski, VT, USA). The mitochondrial potential is expressed as the relative ratio of the mitochondrial J-aggregates (red fluorescence) and the cytoplasmic monomer form of the dye (green fluorescence).

### 2.9 ATP assay

ATP concentration was determined by the commercially available CellTiter-Glo^®^ Luminescent Cell Viability Assay (Promega, Madison, WI) as previously described [11]. The cells were lysed in 100 μl of CellTiter-Glo reagent according to the manufacturer’s recommendations and the luminescent signal was recorded for 1 s on a high sensitivity luminometer (Synergy Mx, Biotek, Winooski, VT, USA). The assay is based on ATP requiring luciferen-oxyluciferin conversion mediated by a thermostable luciferase that generates a stable “glow-type” luminescent signal. ATP standard (dilution series) was used to calculate the cellular ATP amount and the ATP values are shown as percent values of the normoglycemic controls.

### 2.10 Extracellular flux analysis

An XF24 Analyser (Seahorse Biosciences, Billerica, MA) was used to measure metabolic changes in b.End3 cells [10], [41], [42]. The XF24 creates a transient 7 μl chamber in specialised microplates that allows real-time measurement of oxygen and proton concentration changes via specific fluorescent dyes and calculates OCR (oxygen consumption rate) and PPR (proton production rate), measures of mitochondrial respiration and glycolytic activity. The proton production rate is expressed in pMol/min, while ECAR is in pH/min. The OCR and PPR values represent the metabolism of cells, but may also reflect the number of viable cells.

b.End3 cells were exposed to hyperglycemia for 7 days and treated with H_2_S donors for 3 days as described above. The culture medium was changed to unbuffered DMEM (pH 7.4) containing 5 mM glucose, 2 mM L-glutamine and 1 mM sodium pyruvate to allow measurement of the proton production. After determining the basal OCR and PPR values, oligomycin, FCCP and antimycin A were injected through the ports of the Seahorse Flux Pak cartridge to reach final concentrations of 1 μg/ml, 0.3 μM and 2 μg/ml, respectively, to determine the amount of oxygen consumption linked to ATP production, the level of non-ATP-linked oxygen consumption (proton leak) as well as the maximal respiration capacity and the non-mitochondrial oxygen consumption.

### 2.11 Respiratory complex II/III assay

Complex II + III activity was measured by the MitoTox Complex II + III OXPHOS Activity Microplate assay (Abcam, Cambridge, UK). Respiratory complex II (succinate-ubiquinone oxidoreductase) transfers electrons from succinate to Complex III (ubiquinolcytochrome c oxidoreductase) *via* mobile electron shuttle ubiquinone. Complex III transfers electrons to Complex IV (cytochrome c oxidase) *via* mobile electron carrier cytochrome c. The assay measures cytochrome c reduction using succinate as substrate (Complex II + III activity).

Bovine heart mitochondria were used as source of respiratory complexes. Complex I was inhibited by rotenone (10 μM) to block electron transfer from NADH to ubiquinone and Complex IV by potassium cyanide (2 mM) to avoid the reoxidation of cytochrome c. H_2_S donor compounds (10 nM to 10 μM) were mixed with mitochondria (30 μg/ml) in the presence of succinate and oxidised cytochrome c. Cytochrome c reduction was monitored kinetically on a microplate reader (Molecular Devices Spectramax M2e, Sunnyvale, CA) at 550 nm. Mitochondrial complex II/III activity is shown as the maximum velocity of cytochrome c reduction in mOD/min.

### 2.12 Statistics

One-way analysis of variance (ANOVA) was used to detect differences between groups. Post hoc comparisons were made using Tukey’s test. A value of *p* < 0.05 was considered statistically significant. All statistical calculations were performed using Prism 6 analysis software (GraphPad Software, Inc., La Jolla, CA). Data are shown as mean ± SEM values.

## 3 Results

### 3.1 Mitochondria-targeted H_2_S-donor compounds provide controlled H_2_S release

**Fig. 1:**
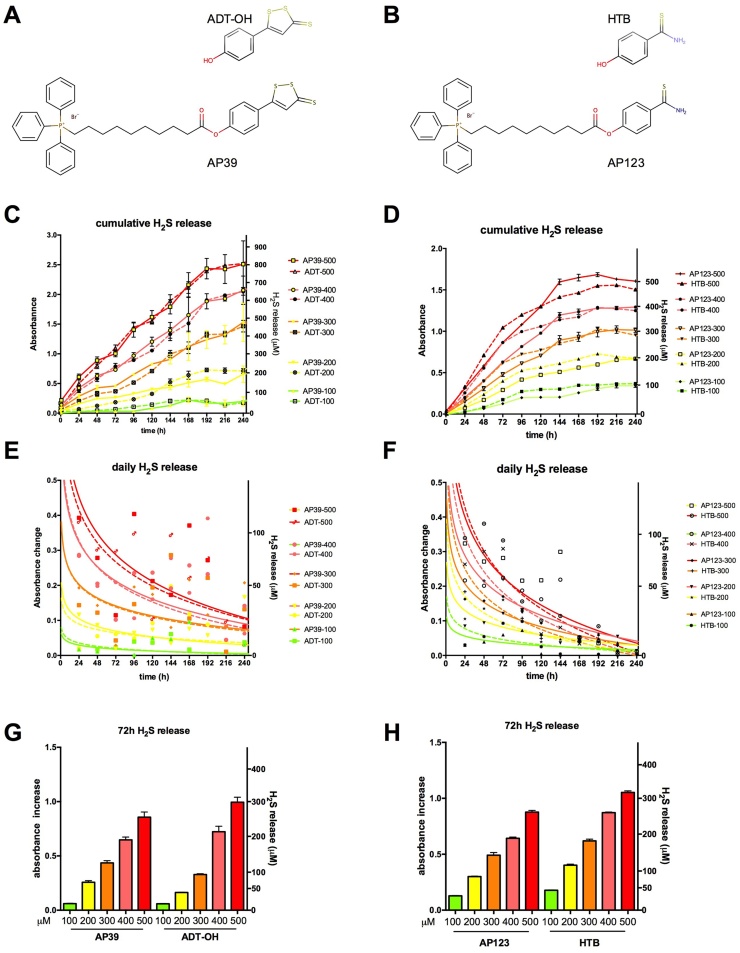
H_2_S release by mitochondrial H_2_S donors. A and B: The chemical structure of mitochondrial H_2_S donors: the H_2_S releasing groups anethole dithiolethione (ADT-OH) in AP39 (A) and 4-hydroxythiobenzamide (HTB) in AP123 (B) are bound by ester linkage to 10-carbon alkyl linker region and the triphenyl phosphonium mitochondrial targeting group. C**-**D: The total amount of H_2_S released from non-mitochondrial (ADT-OH, HTB) and mitochondrial (AP39, AP123) H_2_S donors (100–500 μM) was detected in cell culture medium (DMEM supplemented with 10% FBS) for 10 days. E**-**F: Daily H_2_S release values are plotted with curve-fitting results to highlight the donor compound decomposition. G**-**H: The total amount of H_2_S liberated from mitochondrial and respective non-mitochondrial H_2_S donors over the first 3-day long period is shown.

H_2_S has been proposed as an endogenous antioxidant and plays important roles in inflammatory and vascular diseases. The need for slow-release H_2_S donor compounds was recognised since higher concentrations of the gas are toxic, the half-life of H_2_S is very short and simple salts like sodium hydrosulfide (NaSH) and sodium sulfide (Na_2_S) can only provide instantaneous H_2_S generation [43], [44], [45]. Anethole dithiolethione (ADT-OH) and 4-hydroxythiobenzamide (HTB) represent two simple moieties that release H_2_S slowly. Mitochondrial H_2_S donors (AP39 and AP123) were generated by linking ADT-OH and HTB to a triphenylphosphonium mitochondrial targeting motif *via* a 10-carbon linker region (Fig. 1A and B, Supporting information). This targeting group may result in a 500-fold accumulation of the drug in the mitochondria [46]. Both mitochondrial H_2_S donors and their non-mitochondrial counterparts provide gradual H_2_S production lasting for 7–10 days in cell culture medium (Fig. 1C and D). The mitochondrial targeting group in AP39 does not change the time course of H_2_S release by ADT-OH, but slightly slows down the H_2_S liberation from HTB moiety in AP123 although the mechanism for this is not clear. HTB and AP123 contain a single sulfur atom, thus they can release one H_2_S molecule per donor compound. The expected molar amounts of H_2_S are produced over a 7-day-long period. AP39 and ADT-OH contain 3 sulfur atoms and are possibly capable of higher H_2_S release over 10 days of follow-up. With the shorter period of H_2_S release, a steeper decrease is detectable in H_2_S production for AP123 and HTB than for AP39 and ADT-OH (Fig. 1E and F). While the kinetics are different, the total amount of H_2_S production is comparable during a 3-day long treatment period: approx. 0.6 mol of H_2_S are produced by a mole the H_2_S donors (Fig. 1G and H).

**Fig. 2:**
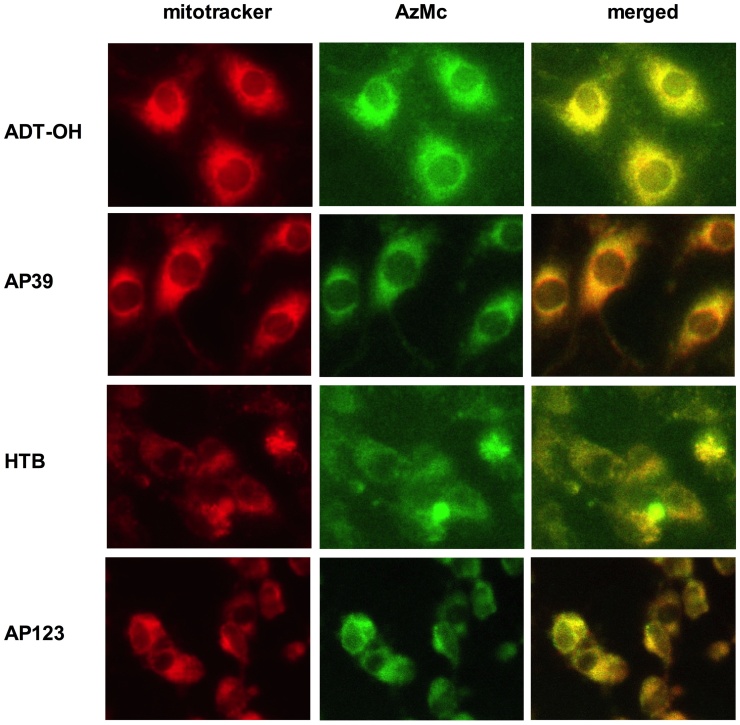
Localization of H_2_S release. b.End3 microvascular endothelial cells were pre-treated with H_2_S donor compounds (30 μM, ADT-OH, AP39, HTB and AP123), then loaded with fluorescent H_2_S sensor AzMc and mitotracker stain. The mitochondria (mitotracker signal) are shown in red and the H_2_S production (AzMc signal) in the cells is shown in green. The H_2_S signal completely overlaps with the mitochondrial signal in mitochondrial H_2_S donor treated cells (as displayed in the merged channels), while in the non-mitochondrial H_2_S donor-treated cells higher non-mitochondrial H_2_S signal is detectable.

We investigated the cellular localisation of H_2_S production following the H_2_S donor administration to confirm that the presence of the mitochondrial targeting group increases the mitochondrial H_2_S release. Endothelial cells treated with the compounds were loaded with fluorescent H_2_S sensor 7-azido-4-methylcoumarin (AzMc) [27] and the H_2_S production was detected by fluorescence microscopy (Fig. 2). Cells treated with the mitochondrial donor compounds showed predominant mitochondrial H_2_S production. While mitochondrial H_2_S generation was evident in all cells, those treated with non-mitochondrial H_2_S donors showed higher presence of extra-mitochondrial H_2_S than those treated with the mitochondrial donors. It has to be mentioned that the ester linkage between the mitochondrial targeting moiety and the H_2_S donor group could be cleaved by cellular esterases increasing the non-mitochondrial H_2_S production in cells treated with AP39 or AP123. However, mitochondrial but not cytoplasmic H_2_S was rapidly detected with each compound suggesting esterase cleavage was minimal.

**Fig. 3:**
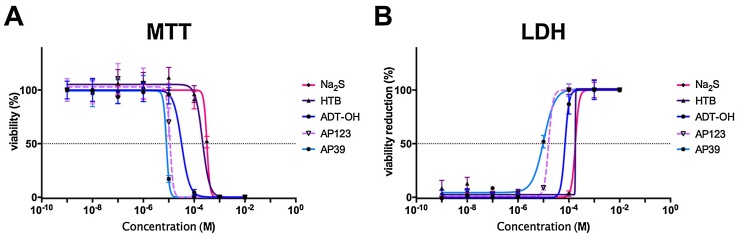
Tolerability of H_2_S donors. b.End3 cells were treated with mitochondrial and non-mitochondrial H_2_S donor compounds for 24 h. A: The cellular viability was measured by the MTT assay. B: LDH release was detected by measuring the LDH activity in the cell culture supernatant. The non-mitochondrial H_2_S donors are better tolerated by the cells: the mitochondrial H_2_S donors reduce the cell survival at lower concentrations.

It is well established that H_2_S causes toxicity at high concentrations by blocking the mitochondrial respiration. This effect is believed to occur *via* inhibition of complex IV (cytochrome c oxidase) [47], [48], [49], but blockage of the mitochondrial respiration may also occur as a consequence of H_2_S-mediated electron donation and reduction of the mitochondrial membrane potential. To test the tolerability of H_2_S donor compounds, we exposed b.End3 endothelial cells to H_2_S donors in a wide concentration range (1 nM–10 mM) and measured the cell survival after 24 h (Fig. 3). All compounds were well tolerated at lower concentrations and induced cell death in a narrow concentration range. Sodium sulfide was tolerated by endothelial cells up to 300 μM but induced cell death above that (TC_50_ = 318.9 μM). The tolerance of HTB was comparable to Na_2_S (TC_50_ = 165.5 μM) while ADT-OH had a lower TC_50_ value (TC_50_ = 69.5 μM) probably due to its higher H_2_S producing capacity. (It has more sulfurs than the other compounds and could release more than one H_2_S per drug molecule). The mitochondria-targeted H_2_S donors caused no toxicity up to 1 μM in endothelial cells and the tolerable concentration was only one order of magnitude lower than their non-mitochondrial counterparts (AP123: TC_50_ = 16.7 μM, AP39: TC_50_ = 7.7 μM). In summary, mitochondrial H_2_S donors are safe to use at sub-micromolar concentrations in endothelial cells.

### 3.2 H_2_S donors inhibit the mitochondrial ROS production in hyperglycemic endothelial cells

High glucose-induced mitochondrial oxidant production plays a central role in mitochondrial dysfunction in endothelial cells [8], [10]. The respiratory chain is primarily responsible for the superoxide generation in the mitochondria in hyperglycemia [8]. Since this process requires electrons, extra protons are left behind and an increase is induced in the transmembrane proton gradient. Mitochondrial hyperpolarisation plays an important part in the increased ROS production in hyperglycemia, since restoration of the mitochondrial potential blocks the mitochondrial superoxide generation [11]. H_2_S acts as an electron donor in the electron transport chain and it is shown to normalise the membrane potential and inhibit the mitochondrial ROS production in hyperglycemia [17]. The instant generation of H_2_S by NaSH or Na_2_S and the small portion of mitochondrial H_2_S produced by these salts requiring their use at high concentrations (and doses) are non-ideal in long-term diseases, thus we tested the ROS-inhibitory effects of mitochondrial slow-release donors AP39 and AP123.

**Fig. 4:**
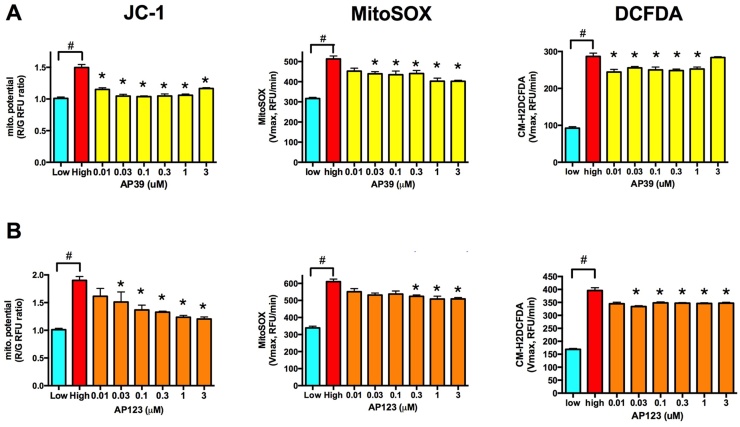
Mitochondrial H_2_S donors protect against ROS production in hyperglycemic endothelial cells. A-B: b.End3 endothelial cells were exposed to high extracellular glucose for 7 days with a single AP39 (A) or AP123 (B) treatment on the 4th day of hyperglycemia. The mitochondrial membrane potential was measured by JC-1, the mitochondrial superoxide production by MitoSOX Red, and the cellular ROS production by CM-H_2_DCFDA. AP39 and AP123 restored the mitochondrial membrane potential and reduced the ROS production. (#p < 0.05 high glucose induced significant increase in mitochondrial membrane potential or ROS production. *p <0.05 H_2_S donor compounds significantly reduced the mitochondrial membrane potential or ROS production compared to hyperglycemic control cells.).

Both AP39 and AP123 significantly reduced hyperglycemia-induced increase in the mitochondrial membrane potential at low nanomolar concentrations (Fig. 4A and D). Both compounds reduced the mitochondrial ROS production as detected by MitoSOX Red (Fig. 4B and E) and also caused a slight decrease in the cellular ROS production as measured by CM-H_2_DCFDA (Fig. 4C and F). AP39 was more effective than AP123 that might be explained by the higher H_2_S release of AP39. It is of note that a single treatment of these mitochondrial donors provided protection over a 3-day-long period at 1000-fold lower concentration than the previously reported cytoprotective concentration of H_2_S using repeated administration [17].

**Fig. 5:**
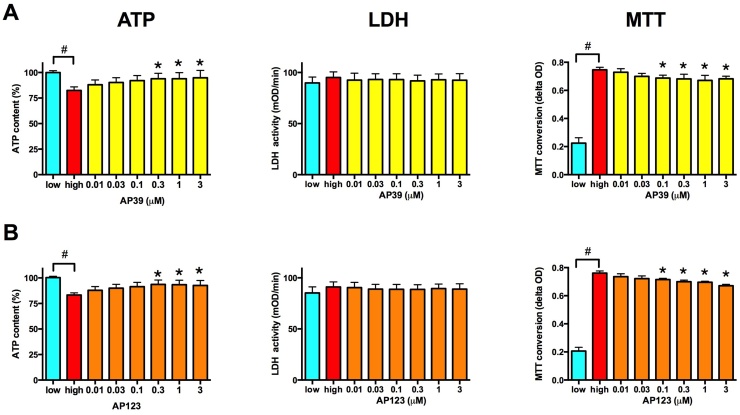
Mitochondrial H_2_S donors reduce the cellular hypermetabolism in hyperglycemic endothelial cells. A-B: b.End3 endothelial cells were exposed to high extracellular glucose for 7 days with a single AP39 (A) or AP123 (B) treatment on the 4^th^ day of hyperglycemia. The MTT reducing capacity, the total cellular LDH activity and the cellular ATP content were measured on the 7th day. (# p < 0.05 high glucose induced significant changes in the cellular MTT reducing capacity and ATP content. * p < 0.05 H_2_S donor compounds significantly reduced the MTT reduction and increased the cellular ATP content.).

Mitochondrial dysfunction affects the cellular energy production in hyperglycemic endothelial cells and results in a decrease in the cellular ATP content after 7 days in b.End3 cells (Fig. 5A and D). Both AP39 and AP123 increased the cellular ATP content in a concentration-dependent manner (Fig. 5A and D) supporting the hypothesis that H_2_S-donor-mediated electron donation increases the mitochondrial ATP production [21]. Hyperglycemia did not induce changes in the cellular LDH activity in b.End3 endothelial cells (Fig. 5B and E), but there was significant increase in the cellular MTT converting capacity (Fig. 5C and F). This increase in the cellular MTT conversion was probably a compensatory activation of the citric acid cycle after long-term exposure to high extracellular glucose. None of the compounds affected the cellular LDH activity (Fig. 5B and E), but both compounds induced a significant decrease in the cellular MTT conversion (Fig. 5C and F).

**Fig. 6:**
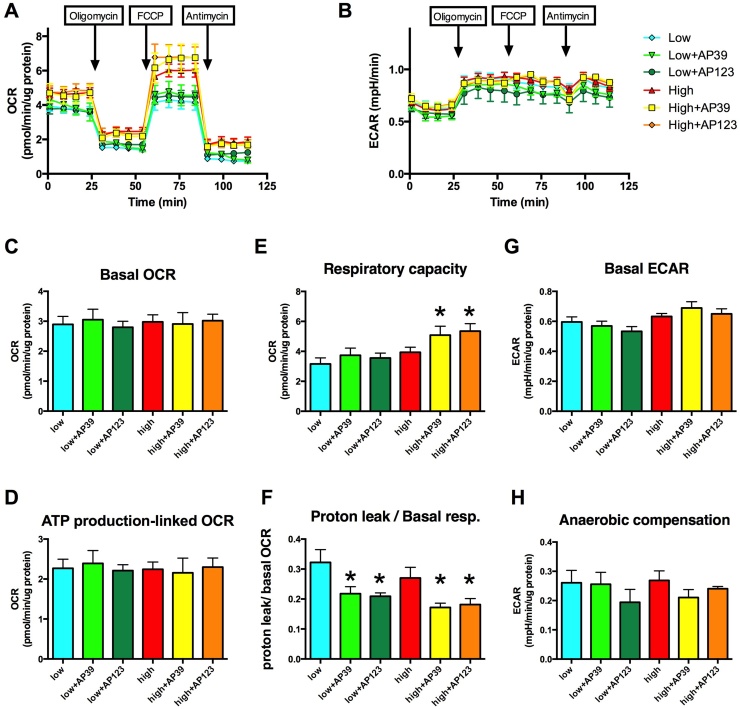
Mitochondrial H_2_S donors affect the cellular bioenergetics. b.End3 cells exposed to 7-day-long hyperglycemia were treated with AP39 (30 nM) or AP123 (100 nM) and the metabolic profile of the cells was studied by extracellular flux analysis. Sequential injections of Oligomycin (1 μg/ml), FCCP (0.3 μM) and antimycin A (2 μg/ml) were used to measure A: the cellular oxygen consumption rate (OCR) and B: the extracellular acidification rate (ECAR). C: Basal oxygen consumption, D: ATP production linked oxygen consumption (determined by oligomycin injection), E: total respiratory capacity (determined following the addition of FCCP) and F: the proton leak/basal respiration was determined. G: Acid production of basal metabolism and H: acid production during anaerobic compensation was determined. AP39 and AP123 increase the respiratory capacity of the cells. (n = 3, *p < 0.05 compared to hyperglycemic control).

To test the effect of the compounds on cellular bioenergetics, we performed metabolic profiling of b.End3 endothelial cells treated with AP39 or AP123 for 3 days using extracellular flux analysis (Fig. 6). Hyperglycemia induced subtle changes in the cellular metabolism at this stage and there is no detectable change in the basal OCR and ECAR (Fig. 6C and G), but the non-mitochondrial oxygen consumption is higher in the hyperglycemic cells: the residual OCR is elevated after blocking the mitochondria with oligomycin, FCCP and antimycin A (Fig. 6A). There was no detectable change in oxygen consumption linked to mitochondrial ATP-production, as measured by ATP synthase inhibition (Fig. 6D), but the mitochondrial H_2_S donors induced significant increase in the respiratory capacity (Fig. 6E) that is in line with prior results showing that increased intra-mitochondrial H_2_S production affects this measure [21]. The mitochondrial H_2_S donors improve the coupling efficiency and significantly reduce the proton leak (Fig. 6F) that can explain the increased cellular ATP content in the cells (Fig. 5A and D) without a measurable increase in oxygen consumption. There is no change in the anaerobic metabolism in cells treated with mitochondrial H_2_S donors (Fig. 6G) that further confirms that the compounds do not inhibit mitochondrial respiration at low nanomolar concentrations. The predominantly mitochondrial localisation (Fig. 2**)** strongly suggests that there was no interference with anaerobic compensation following the inhibition of mitochondrial respiration (Fig. 6H).

**Fig. 7:**
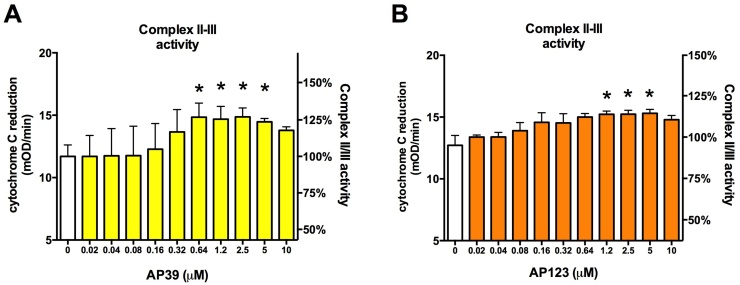
Mitochondrial H_2_S donors increase the respiratory Complex II/III activity. A and B: Cytochrome c reduction was monitored in bovine heart mitochondria following Complex I and IV blockade by rotenone and KCN, respectively. A: AP39 was added at 10 nM–10 μM and complex II/III activity was measured kinetically, B: Mitochondria were treated with AP123 (10 nM–10 μM) and the respiratory complex activity was monitored. (*p < 0.05, H_2_S donors significantly increased the respiratory complex activity).

Mitochondrial H_2_S oxidation is a complex process that requires three enzyme activities: 1) sulfide-quinone oxidoreductase (SQR) catalyses the two-electron oxidation of H_2_S to the level of elemental sulfur by simultaneously reducing a cysteine disulfide such that a persulfide group is formed, 2) sulfur dioxygenase oxidises persulfides to sulfite, consuming molecular oxygen and water and 3) sulfur transferase produces thiosulfate by transferring a second persulfide from SQR to sulfite [22]. During the first step of H_2_S oxidation, the electrons are fed into the respiratory chain via the quinone pool (at the level of complex III). Oxygen consumption occurs only through the second step of H_2_S oxidation, thus feeding of electrons from H_2_S to the respiratory system does not necessarily increase the cellular oxygen consumption. To confirm that the action of mitochondrial H_2_S donors increase the electron transfer, we performed a Complex II/III activity assay (Fig. 7). We blocked input from Complex I by rotenone and inhibited cytochrome c oxidation (Complex IV) by potassium cyanide. In the presence of substrate (succinate) Complex II transfers electrons to ubiquinone and Complex III to cytochrome c. The rate of cytochrome c reduction was measured in the absence or presence of AP39 or AP123. Both compounds induced a concentration-dependent increase in complex III activity at concentrations below 2.5 μM (Fig. 7A and B), but a decrease was detected at higher concentrations (5–10 μM). AP123 induced similar changes to AP39 but at twice as high concentration possibly due to its lower H_2_S producing capacity. These results confirmed that the compounds directly affected the respiratory complex activities.

## 4 Discussion

The positive effects of H_2_S supplementation in diabetes were confirmed by several studies but long-term administration of H_2_S remained a challenging issue [17], [26], [50], [51]. H_2_S is volatile and has short half-life *in vivo*, thus for long-term treatment its preferable to use donor molecules (prodrugs) that release H_2_S at a controlled rate. Several H_2_S donor compounds have been developed over the last couple of years and various H_2_S producing chemistries have been implicated but the control of H_2_S generation is still not perfect [43], [52], [53]. A further problem may arise from the side effects caused by the by-products that are formed during H_2_S release, thus in chronic diseases it is necessary to reduce the concentration of the donors as much as possible since very long treatment periods are anticipated. One option is to deliver the H_2_S donors to specific cell types or subcellular compartments to minimise the off-target effects. The subset of cell types, that are involved in diabetic complications and should benefit from H_2_S supplementation, includes capillary endothelial cells, mesangial cells, neurons and Schwann cells in peripheral nerves [8]. The glucose-induced damage is orchestrated by the mitochondria via superoxide generation that promotes all other oxidative stress pathways in diabetes [8], thus mitochondrial oxidant production is the foremost target in the cells.

It is difficult to determine the mitochondrial concentration of H_2_S that might be associated with beneficial effects in the cells and various methodologies produced strikingly different results, but the amount to produce stimulatory effect on cellular bioenergetics is probably between 6 nM and 1 μM [21], [54], [55]. In contrast, a ∼1000-fold higher concentration (100–300 μM exogenous H_2_S) is needed to normalise the mitochondrial membrane potential and decrease the oxidant production in endothelial cells exposed to high glucose concentrations, presumably because the H_2_S was not targeted to mitochondria [17]. Extracellular consumption of H_2_S, extra-mitochondrial metabolism and low penetration might also contribute to this huge difference. The amount of H_2_S that blocks complex IV and has inhibitory effect on the respiration is no more than 1 order of magnitude higher than its stimulatory concentration [21], [48], [54], [55] thus dosing can be challenging. Furthermore, it is unclear whether exogenous H_2_S supplementation affects the endogenous H_2_S production and whether the concentrations determined by prior assays truly reflect the beneficial amount of H_2_S on the long term. Overall, prior results suggest that mitochondria-specific delivery of H_2_S can greatly reduce the therapeutic concentration of H_2_S donors. We found that AP39 and AP123 were effective against hyperglycemic injury at >1000-fold lower concentrations than Na_2_S in endothelial cells. The cytoprotective concentrations of the compounds (30–300 nM) are similar to the values previously reported for AP39 [34], [35], [56], [57]. The mitochondrial potential normalising and antioxidant effects of the compounds also confirm that H_2_S-mediated cytoprotection depends on its mitochondrial effect in hyperglycemic endothelial cells. It is unlikely that the mechanism of protection by mitochondrial-targeted H_2_S is by, or includes, upregulation of H_2_S synthesising enzymes as this has not been previously observed [58].

The triphenylphosphonium targeting moiety of AP39 and AP123 provides potential–dependent drug accumulation in the mitochondria [46] and also assures that H_2_S concentration is kept within a safe range since normalisation of the mitochondrial potential will reduce the drug accumulation. On the other hand, while the mitochondrial membrane potential is elevated the intra-mitochondrial drug concentration will be higher than in cells with normal or reduced mitochondrial potential at a given loading concentration of the drug. Also, in metabolically active cells the high consumption of H_2_S will not result in a drop in H_2_S donors, since mitochondria will be replenished with new donor molecules by the re-equilibration process and a relatively stable supply of H_2_S will be maintained by the use of these donor compounds.

The antioxidant effect of AP39 and AP123 are comparable, but the effective concentration of AP39 is slightly lower than that of AP123 (Fig. 4**)**. AP39 also induced an increase in complex II/III activity at a slightly lower concentration than AP123 that supports higher mitochondrial H_2_S release by AP39 (Fig. 7**)**. Both AP39 and AP123 provide H_2_S release for multiple days but AP39 is capable of releasing more H_2_S than AP123 (Fig. 1**)**. The higher H_2_S release by AP39 is also evidenced by its lower toxic concentration: AP39 has a TC_50_ of 7.8 μM while AP123’s TC_50_ is 16.7 μM (Fig. 3**)**; concentrations far exceeding their cytoprotective concentration range (*e.g.* 10–300 nM, Fig. 3, Fig. 4, Fig. 5, Fig. 6, Fig. 7). The toxic concentration of the non-mitochondrial H_2_S donors ADT-OH and HTB is ten times higher (69.5 μM and 165.5 μM, respectively) than their mitochondrial counterparts. The ten-fold increase in the mitochondrial H_2_S delivery achieved by the ester-linked mitochondrial targeting moiety possibly suggests ten times lower risk of side effects caused by the metabolites of the drugs. The molecular mechanism of H_2_S release from 1,2-dithiole-3-thione compounds are still unclear [59], but the mitochondrial redox environment may affect this process. Furthermore, H_2_S generation from ADT-OH or AP39 can occur through multiple steps and each of these steps may be affected by various metabolites in the mitochondria. On the other hand, HTB compounds are more likely to liberate H_2_S through a single step that is not affected by the metabolites, possibly allowing for better control of H_2_S generation. Interestingly, HTB is the chosen H_2_S donor moiety in many novel H_2_S-releasing therapeutics including various non-steroidal anti-inflammatory drugs (NSAIDs) and some of them (*e.g.* the naproxen derivative ATB-346) already reached clinical trial phases [52].

H_2_S supplementation using natural products may represent an alternative approach for long-term treatment. Garlic is the most commonly used sulfur-rich nutrient that can provide H_2_S using it either freshly or its extract as a dietary supplement. Allicin (diallyl thiosulfinate), the main source of H_2_S in garlic, decomposes to various sulfur-containing compounds in aqueous solutions including DADS and DATS [33], [53], [59]. DADS and DATS release H_2_S in a thiol-dependent manner and they may deplete the cellular glutathione pool [60], [61], [62]. While this chemical approach may help control the H_2_S release, the loss of glutathione increases the risk of oxidative damage in a pro-oxidant state like diabetes and H_2_S toxicity was also associated with it [63]. Interestingly, the opposite effect of DADS, an increase in the cellular glutathione level was also reported after prolonged treatment periods [64] that may be caused by H_2_S produced from the donors, since H_2_S itself increases the glutathione concentration [65]. However, if this is the case the elevated glutathione content should result in further H_2_S generation from thiol-dependent donors causing fluctuations in the H_2_S levels and making the dosing more complicated than with the HTB or ADT-OH-based donors. While the beneficial effects of garlic in diabetes models were confirmed by multiple studies [29], [30], [31], [66], garlic had no effect on endothelial function and oxidative stress in diabetic patients in a recent pilot trial and only little increase was detected in the glutathione level [67], which further support the difficulties with dosing of garlic-based dietary supplements.

## 5 Conclusion

Mitochondrial slow release H_2_S donors provide protection against the prolonged low level oxidative stress induced by hyperglycemia in endothelial cells. They increase the electron transfer rate at respiratory complex III and have beneficial effect on cellular bioenergetics. These compounds offer the potential to be much safer than inorganic sulfide salts (Na_2_S or NaSH) and target delivery to mitochondria: the concentration of AP39 and AP123 that results in these positive effects are >2 orders of magnitude lower than their maximum tolerated concentration *in vitro*, whereas the cytoprotective concentration of inorganic sulfide salts is very close to their toxic concentrations [17]. Furthermore, the slow H_2_S release in biological buffers combined with high lipophilicity and mitochondria-targeting allows fewer drug administrations making these compounds preferable to previously used H_2_S donors.

## Conflicts of interest

MW, MEW and the University of Exeter have intellectual property (patent filings) on slow release hydrogen sulfide donors including AP39, AP123, related compounds and their use.

## Author contributions

DG and MW designed the experiments. DG, MEW, RT, SL, JLW, and AP conducted the experiments. DG and MW analysed the data and wrote the first draft of the manuscript. All authors contributed to proof reading and manuscript revision.
