# Supplementary material for: The novel mitochondria-targeted hydrogen sulfide (H2S) donors AP123 and AP39 protect against hyperglycemic injury in microvascular endothelial cells in vitro
Source: Pharmacol Res. 2016 Nov;113(Pt A):186–98. doi: 10.1016/j.phrs.2016.08.019 (PMC5113977; doi:10.1016/j.phrs.2016.08.019)

General experimental and instrumentation details and spectra for AP123

General information

For general information regarding synthetic and analytical chemistry methods, see reference **28**. (Le Trionnaire S, Perry A, Szczesny B, Szabo C, Winyard PG, Whatmore JL, et al. The synthesis and functional evaluation of a mitochondria-targeted hydrogen sulfide donor, (10-oxo-10-(4-(3-thioxo-3H-1,2-dithiol-5-yl)phenoxy)decyl)triphenylphosphonium bromide (AP39). Med Chem Commun. 2014; (5):728-36. doi: 10.1039/C3MD00323J.)

AP123 high-resolution mass spectrum:

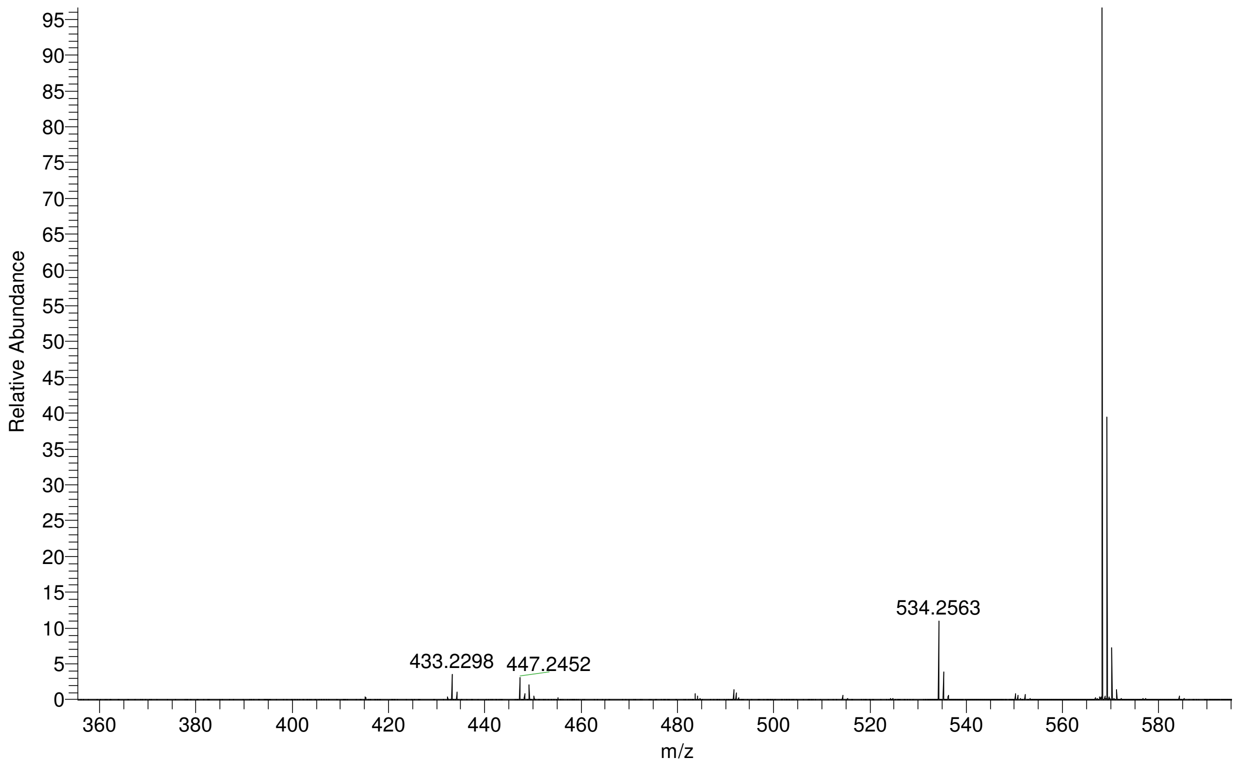

AP123 infrared spectrum (KBr disc):

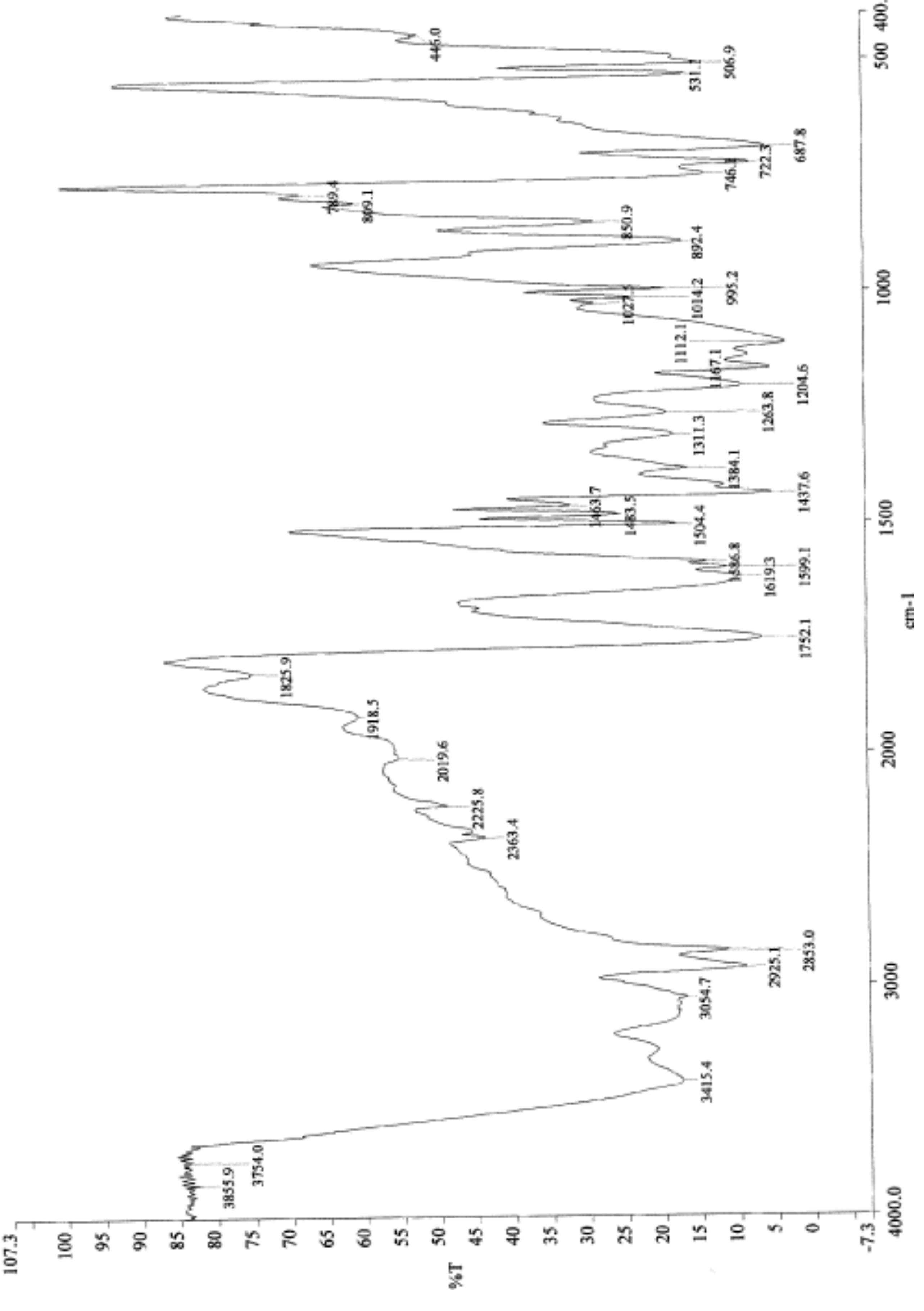

AP123 ^{1}H NMR spectrum (300 MHz; $CDCl_3$):

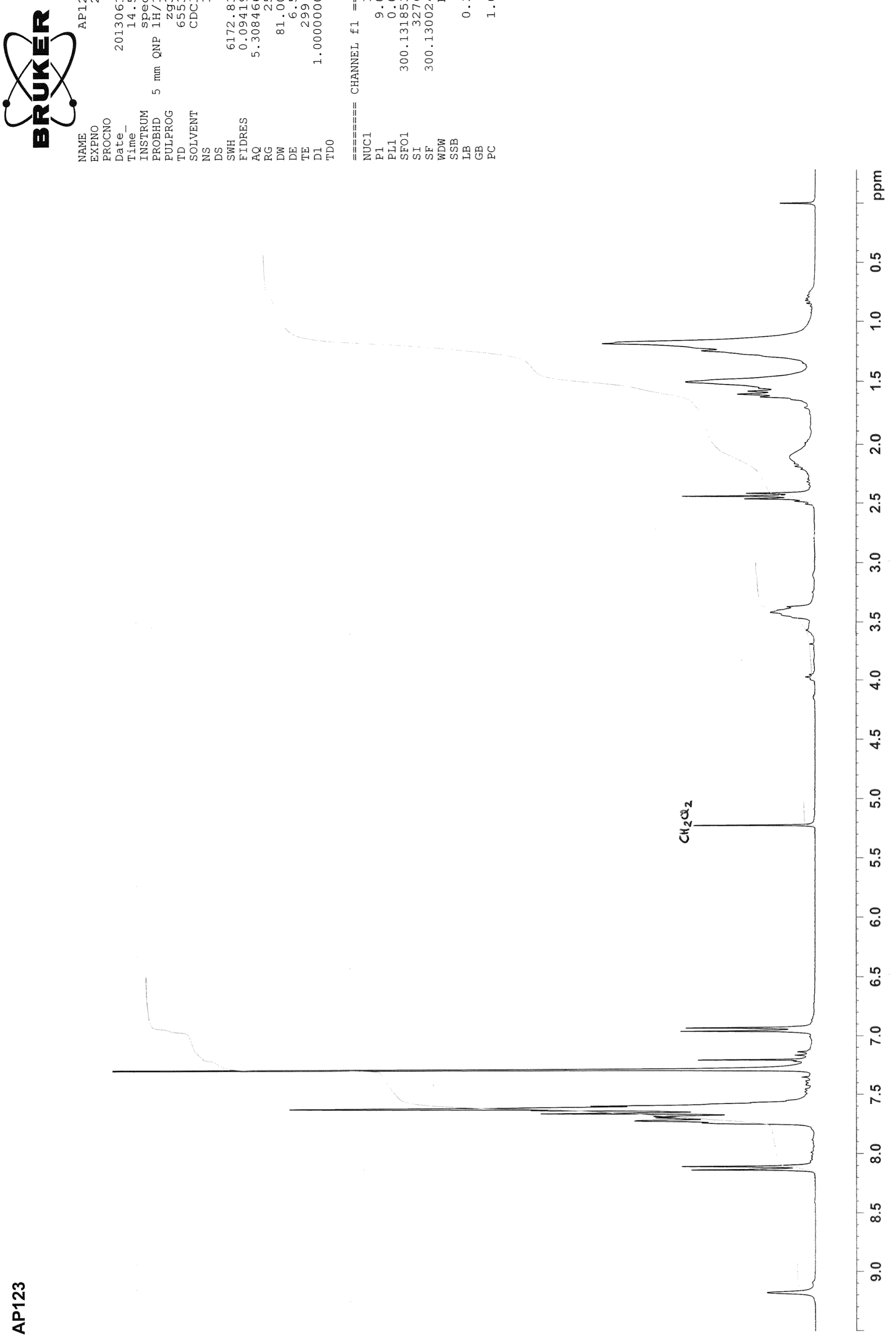

AP123 ^{31}P NMR spectrum (121 MHz; $CDCl_3$):

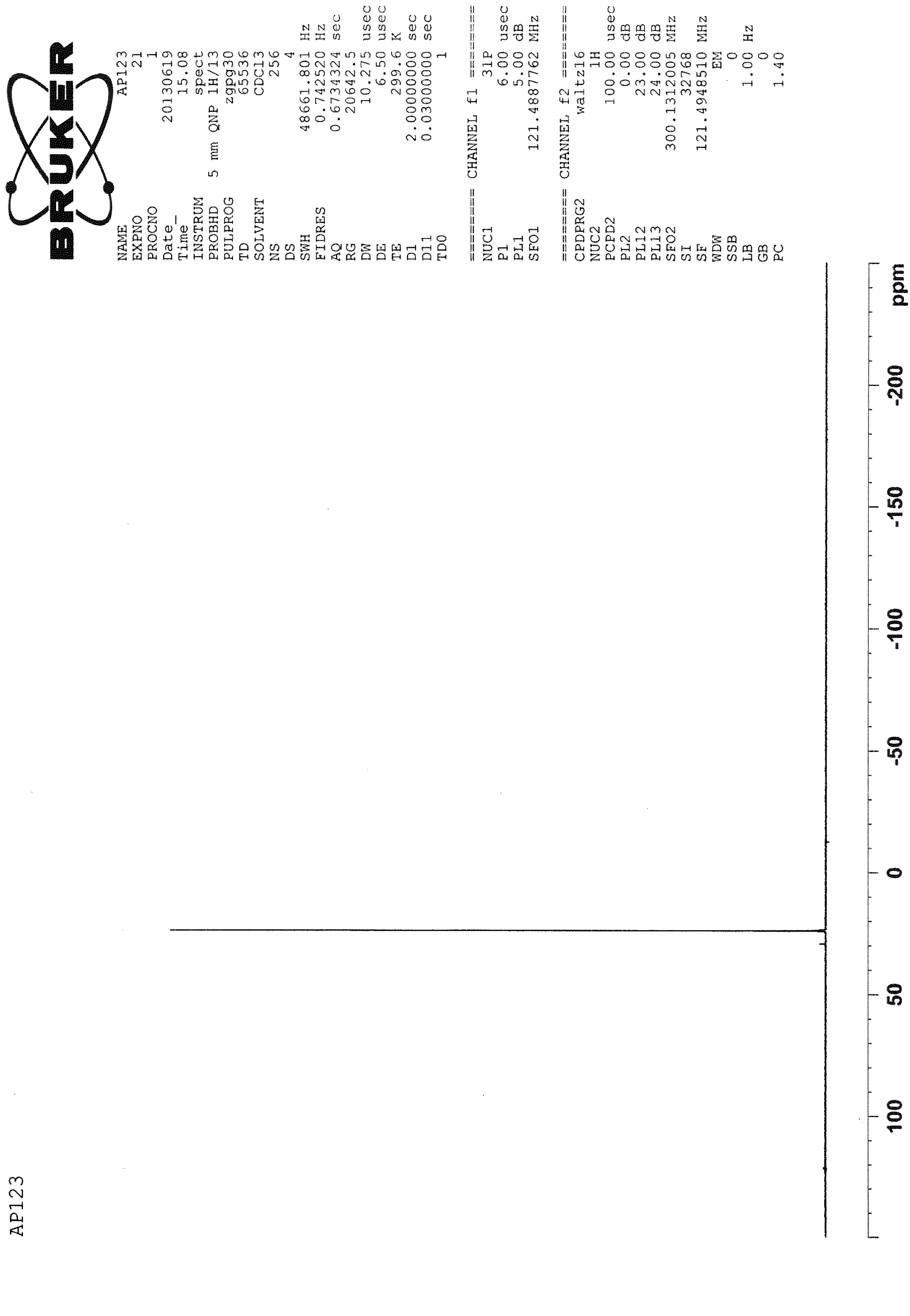

AP123 ^{13}C NMR spectrum (75 MHz; $CDCl_3$):

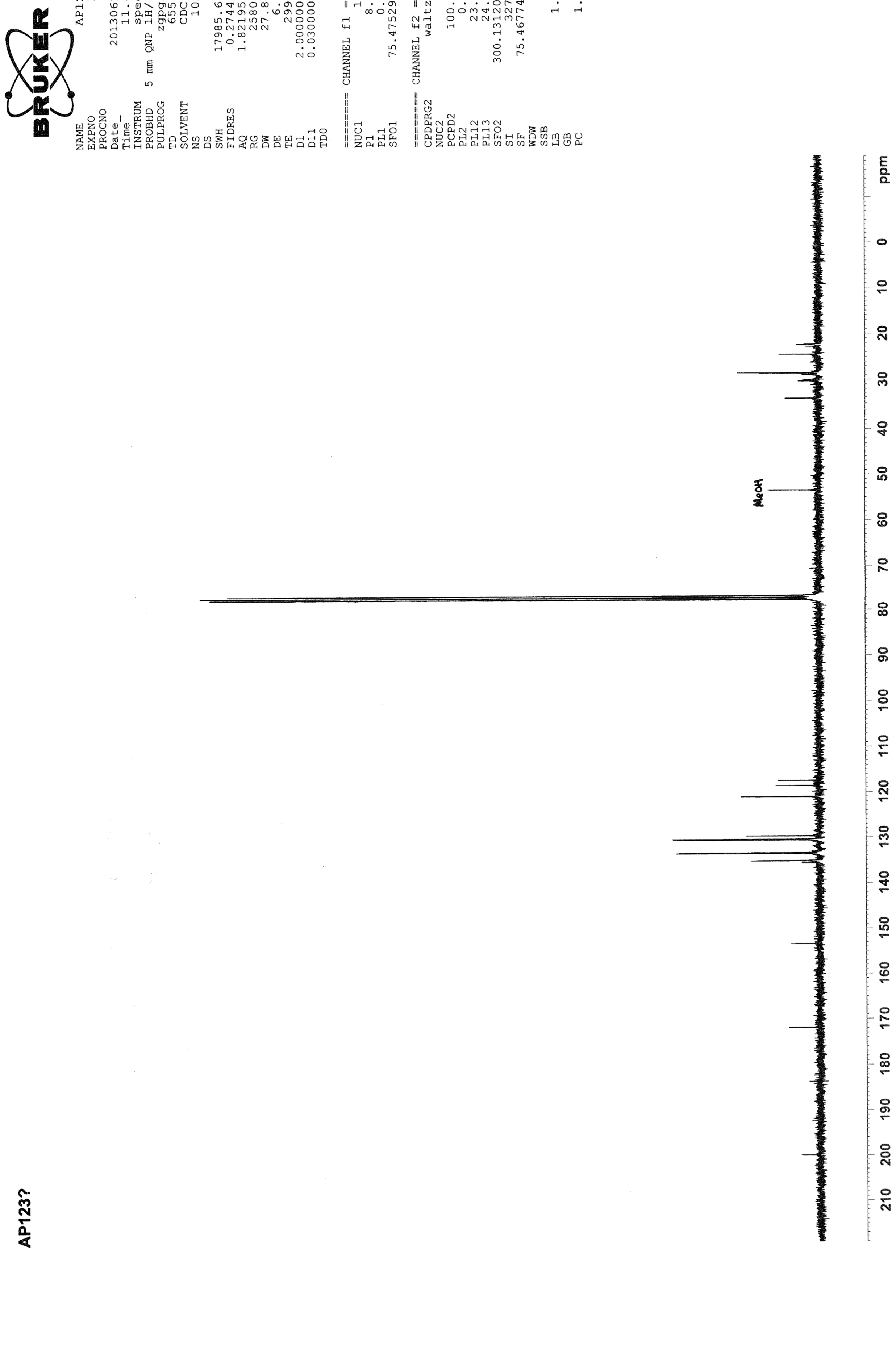

Supplement: Supplementary file 1 [file mmc1.pdf]
